# Generation of NK cells with chimeric-switch receptors to overcome PD1-mediated inhibition in cancer immunotherapy

**DOI:** 10.1007/s00262-022-03317-y

**Published:** 2022-11-10

**Authors:** Katharina H. Susek, Ysabel A. Schwietzer, Maria Karvouni, Mari Gilljam, Marton Keszei, Alamdar Hussain, Johan Lund, Muhammad Kashif, Andreas Lundqvist, Hans-Gustaf Ljunggren, Hareth Nahi, Arnika K. Wagner, Evren Alici

**Affiliations:** 1 Department of Medicine Huddinge, Center for Hematology and Regenerative Medicine, Karolinska Institutet, Karolinska University Hospital Huddinge, Stockholm, Sweden; 2 Department of Microbiology, Tumor and Cell Biology, Karolinska Institutet, Stockholm, Sweden; 3 Department of Oncology-Pathology, Karolinska Institutet, Solna, Stockholm, Sweden; 4 Department of Medicine, Center for Infectious Medicine, Karolinska Institutet, Karolinska University Hospital Huddinge, Stockholm, Sweden

**Keywords:** Antibody-dependent cellular cytotoxicity, Chimeric switch receptor, Hematologic neoplasms, Immunotherapy, Natural killer cells, Programmed cell death 1 receptor

## Abstract

Multiple myeloma (MM) is an incurable hematological cancer, in which immune checkpoint inhibition (ICI) with monoclonal antibodies (mAbs) has failed due to uncontrollable immune responses in combination therapies and lack of efficacy in monotherapies. Although NK cell-specific checkpoint targets such as NKG2A and KIRs are currently being evaluated in clinical trials, the clinical impact of NK cells on the PD1 cascade is less well understood compared to T cells. Furthermore, while NK cells have effector activity within the TME, under continuous ligand exposure, NK cell dysfunctionality may occur due to interaction of PD1 and its ligand PD-L1. Due to above-mentioned factors, we designed novel NK cell specific PD1-based chimeric switch receptors (PD1-CSR) by employing signaling domains of DAP10, DAP12 and CD3ζ to revert NK cell inhibition and retarget ICI. PD1-CSR modified NK cells showed increased degranulation, cytokine secretion and cytotoxicity upon recognition of PD-L1^+^ target cells. Additionally, PD1-CSR^+^ NK cells infiltrated and killed tumor spheroids. While primary NK cells (pNK), expressing native PD1, showed decreased degranulation and cytokine production against PD-L1^+^ target cells by twofold, PD1-CSR^+^ pNK cells demonstrated increased activity upon PD-L1^+^ target cell recognition and enhanced antibody-dependent cellular cytotoxicity. PD1-CSR^+^ pNK cells from patients with MM increased degranulation and cytokine expression against autologous CD138^+^PD-L1^+^ malignant plasma cells. Taken together, the present results demonstrate that PD1-CSR^+^ NK cells enhance and sustain potent anti-tumor activity in a PD-L1^+^ microenvironment and thus represent a promising strategy to advance adoptive NK cell-based immunotherapies toward PD-L1^+^ cancers.

**Supplementary Information:**

The online version contains supplementary material available at 10.1007/s00262-022-03317-y.

## Background

The field of cancer immunotherapy has shown breakthrough advances due to the success of immune checkpoint inhibition (ICI) and chimeric antigen receptor (CAR)-T cell therapy. As resistance toward ICI and adoptive cell therapies occurs, combination therapies are explored [1, 2]. One approach is to genetically modify effector cells to make them less prone to PD-L1/PD-L2-mediated inhibition. Currently, several registered clinical trials employ PD1 knockout (PD1-KO) or PD1 disrupted chimeric antigen receptor (CAR) T cells for various malignancies [3–5]. Although this approach has been proven successful in some tumor models, emerging data indicate that PD1-KO might also impair T cell functionality [6]. Therefore, another novel approach is the utilization of chimeric switch receptors (CSR) that link PD-L1 engagement to an activating signal.

Natural killer (NK) cells are innate lymphoid cells that recognize and kill infected, stressed or malignant cells without prior antigen exposure [7]. They exert direct cytotoxicity against target cells and enhance immune responses via cytokine and chemokine secretion [8]. NK cell activation depends on the balance of several germline-encoded inhibitory and activating receptors [9]. One of the strongest activating receptors is CD16 that binds to the constant region (Fc) of immunoglobulins and induces antibody-dependent cellular cytotoxicity (ADCC). Many activating receptors lack a signaling domain and rather depend on adaptor proteins for a functional response. The most prominent of these are the immunoreceptor tyrosine-based activating motif (ITAM)-bearing adaptor proteins CD3ζ and DAP12 as well as DAP10 which signals via a YINM motif [9–11].

In a recent clinical trial, CD19-CAR-NK cells displayed a good clinical response with seven out of eleven patients reaching a complete remission with only minimal toxicity [12]. Adoptive cell therapies, employing NK cells, are thus increasingly becoming important due to several reasons such as a beneficial risk profile [13]. However, there are still many open questions to ensure the success of NK cell-based immunotherapies in the clinical setting. In this study, we addressed the concern of NK cell hypofunctionality due to immune-checkpoint receptor engagement in the tumor microenvironment (TME). Although the role of PD1 on NK cells from healthy individuals is not fully understood, it has been shown that tumor-infiltrating NK cells often show increased PD1 expression with reduced effector cell functionality that can be reverted by PD1-PD-L1 blockade with mAb [14–18]. Therefore, we set out to assess the ability of PD1-based CSR to sustain the functionality of NK-92 and primary NK (pNK) cells against different PD-L1^+^ tumor targets. Here, we demonstrate that PD1-CSR expressing NK-92 and primary NK (pNK) cells increase degranulation, cytokine secretion, and tumor cell killing upon recognition of PD-L1.

## Methods

### Cells

All cell lines were purchased from ATCC. The B cell lymphoma cell line Raji (ATCC® CCL-86™) and the renal cell carcinoma cell line 786-O (ATCC® CRL-1932™) were maintained in RPMI medium (Gibco), supplemented with 10% FBS (Gibco). NK-92 cells (ATCC® CRL-2407™) were maintained in SCGM (CellGenix), supplemented with 20% FBS (Gibco). Cell lines were split every 2–3 days. Interleukin-2 (R&D) was added at a final concentration of 500 U/ml to the cell culture medium of NK-92 cells.

### PBMC and primary NK cell isolation and culture

Peripheral blood mononuclear cells (PBMCs) were obtained from buffy coats. According to institutional guidelines ethical permits were not required for healthy donors due to de-identification of donors. Ethical permits were granted for work with patient derived PBMCs and bone marrow samples (Permit Numbers: 2019-04973 and 2020-02119). PBMC isolation was performed with LymphoPrep™ (Fresenius Kabi) according to the manufacturer’s recommendations. Isolated PBMCs were cultured in SCGM medium (CellGenix), supplemented with 5% human serum (Biowittaker). CD3 Ab (Miltenyi, clone OKT3) was added to the culture at a final concentration of 10 ng/ml on the day of isolation. Interleukin (IL)-2 (R&D) was added to the culture at a final concentration of 500 U/ml on days 1 to 4 (daily), and then five times/week. pNK cells were isolated from PBMCs by negative selection and magnetic separation according to the manufacturer’s recommendations (Miltenyi, 130-092-657). pNK cells were cultured in SCGM medium (CellGenix), supplemented with 10% human serum. IL-21 (ImmunoTools) was added on the day of isolation at a concentration of 20 ng/ml. IL-2 (R&D) was added daily to the culture at a final concentration of 1,000 U/ml.

### Isolation of bone marrow mononuclear cells (BM MNC)

Bone marrow aspirates were obtained from patients with MM after obtaining informed consent and according to our ethical permit (Permit Numbers: 2019-04973 and 2020-02119). BM MNC were isolated using Ficoll-Paque (Sigma-Aldrich) according to the manufacturer’s recommendations. Isolated BM MNC were passaged in RPMI medium (Gibco), supplemented with 10% FBS (Gibco).

### Generation of chimeric switch receptors

For the generation of PD1-based CSR, the canonical human cDNA sequence was used without further modification or codon optimization. A truncated version of PD1 (amino acid (AA) 1–211), which lacks the intracellular signaling domains, was designed and is hereafter referred to as PD1_EC-TM_ CSR. The PD1-CD28-CD3ζ CSR consists of AA 1–170 of PD1, an 85AA long hinge region, the AA sequence 153–220 of CD28 and AA 52–164 of the CD3ζ protein. For the PD1-NKp46 CSR, PD1 AA 1–170 was fused together with AA 239–304 of the NKp46 protein. NKp46 lacks an intracellular signaling domain but can associate with ITAM-bearing CD3ζ homodimers or CD3ζ/FcεRIγ heterodimers through oppositely charged residues within the transmembrane region [19]. In the PD1_Ec_DAP10_TM-IC_ and PD1_Ec_DAP12_TM-IC_ constructs, PD1 protein from AA 1 to 170 was fused to the full length DAP10 (AA 19–93) or DAP12 (AA 22–113) protein. In the PD1_EC-TM_DAP10_IC_ and PD1_EC-TM_DAP12_IC_ CSR the PD1 AA sequence 1 to 212 was fused together with AA 77–93 of DAP10 or AA 73–113 of DAP12. The designed constructs were cloned into the LeGoiG2 or LeGo_T2A-eGFP vector, upstream of the IRES or T2A under the control of the SFFV promoter. The LeGo_T2A-eGFP vector was designed by replacing the IRES of the LeGoG2 vector with a T2A sequence (LeGO-iG2 and LeGo-G2 were a kind gift from Dr B. Fehse) [20]. These plasmids were used to produce VSV-G-pseudotyped lentiviral vectors.

### Generation of PD-L1^+^ and PD-L1^−^ target cell lines

Raji cells were transfected with PD-L1 plasmid (GenScript, OHu22144), using the Amaxa Cell Line Nucleofector kit V (Lonza, VCA-1003) according to the manufacturer’s recommendations. PD-L1 protein was knocked out in 786-0 cells with CRISPR-Cas9 technology according to previously published protocols [21]. Two different guide RNAs were used to generate KO1 (guide RNA sequence: TACCGCTGCATGATCAGCTATGG) and KO2 (guide RNA sequence: TACCATACTCTACCACATATAGG) to ensure obtained results were due to PD-L1 KO and not unwanted off-target alterations associated with CRISPR technology.

### Production of lentiviral vectors

Lentiviruses were generated by calcium-phosphate based transfection (Sigma, CAPHOS-1KT) of either 1 × 10^6^ HEK293FT cells (CRISPR plasmids) or 14 × 10^6^ HEK293FT cells (PD1-CSR plasmids) according to the manufacturer’s recommendations. Briefly, the plasmids of interest were co-transfected with the envelope plasmid pCMV-VSV-G [22] and two packaging plasmids, pDMLg/pPRE [23] and pRSV-Rev [23] to produce VSV-G-pseudotyped lentiviruses. For the transduction of NK cells, lentiviruses were concentrated prior to freezing with the Lenti-X™ concentrator according to the manufacturer’s recommendations (Takara Bio, 631,232). All lentiviruses were titrated on HEK293FT cells. Briefly, 5 × 10^4^ cells per well of a 24-well plate were seeded in medium, containing different amounts of concentrated virus, in the presence of 8 μg/μl protamine sulfate (Sigma-Aldrich P3369-10G). Cells were spinoculated for one hour at 1000 × g and 32 °C after which incubation for 6 h at 37 °C and 5% CO_2_ followed. Protamine-sulfate containing medium was then replaced with fresh medium. Green fluorescent protein (GFP) percentage was analyzed by flow cytometry three days post-transduction. Lentiviral titer was calculated with the formula $\frac{\% \textit{of GFP positive cells} \times 50.000}{{\textit{viral supernatant in ml}}}$

### Lentiviral transduction of NK-92, pNK cells and 786-O cells

NK cells were transduced as described previously [24]. pNK cells were isolated from healthy donor PBMCs. Briefly, cells were seeded at 5 × 10^5^ cells/ml in viral supernatant at an MOI of 4 (NK-92) or 15 (pNK) in the presence of (5Z)-7-Oxozeanol (Biotechne​) and 8 μg/μl protamine sulfate (Sigma-Aldrich P3369-10G). Cells were spinoculated for one hour at 1,000 × g and 32 °C after which incubation for five hours at 37 °C and 5% CO_2_ followed. Protamine-sulfate containing medium was then replaced with fresh medium, containing IL-2 at a final concentration of 500 U/ml (NK-92) or 1000 U/ml (pNK) (R&D 202-IL-500). 786-0 cells were plated at a density of 3,300 cells/cm^2^ in the presence of viral supernatant and 8 μg/μl protamine sulfate (Sigma-Aldrich P3369-10G). Cells were spinoculated for one hour at 800× g and 32 °C after which incubation for five hours at 37 °C and 5% CO_2_ followed. Medium was then replaced with fresh medium. GFP, PD1 and PD-L1 expression were analyzed three days after transduction. Cells were sorted using BD FACS AriaFusion.

### Flow cytometry

The following mAb were used for flow cytometry analysis: CD56 (clone NCAM1), CD16 (clone 3G8) CD3 (clone SK3), CD11b (clone ICRF44), CD14 (clone MOP9) PD1 (clone EH12.1), PD-L1 (clone MIH1), PD-L2 (clone MIH18), CD138 (clone MI15) MICA/B (clone 6D4), CD155 (clone TX24), NKG2A (clone 131,411), NKp44 (clone p44-8), TIM3 (clone 7D3), TIGIT (clone 741,182), DNAM1 (clone DX11) from BD Biosciences; HLA-ABC (clone W6/32), CD38 (clone HIT2), NKp30 (clone p30-15), NKp46 (9E2/NKp46), LAG3 (clone 11C3C65), NKG2D (clone 1D11), CD112 (clone TX31) from BioLegend; CD158a/h/g (clone HP-MA4), CD158e (clone DX9) from Thermofisher, CD158B (clone GL183) from Invitrogen, ULBP256 (clone 165,903) from R&D. All antibodies were titrated prior to usage. Briefly, cells were collected and washed once in PBS. Cells were stained with Aqua live dead cell staining (ThermoFisher) for 20 min at 4 °C, in the dark. Cells were washed once with PBS, containing 2% FBS. Surface staining was performed for 25 min at 4 °C, in the dark. Cells were washed with PBS, containing 2% FBS, centrifuged and fixed with 1% paraformaldehyde for 10 min. Acquisition was performed the following day with Beckman Coulter Cytoflex flow cytometers. Analysis was performed with FlowJo analysis software version 10. Gates were — unless otherwise specified – placed based on the unstained control or FMO (fluorescence minus one).

### NK cell degranulation assay and evaluation of IFNγ and TNF intracellular staining

0.03 × 10^6^ 786-O cells were seeded in a flat 96-well plate 24 h before the assay to allow cells to attach. 0.15 × 10^6^ NK-92 or pNK cells were co-incubated with either 0.15 × 10^6^ Raji cells or 786-O cells in a final volume of 200 μl at 37 °C and 5% CO_2_ for four hours in the presence of CD107a antibody (BioLegend, clone H4A3). Where indicated, Rituximab was added to the co-culture at a final concentration of 2.5 μg/ml. As controls, 0.15 × 10^6^ NK-92 or pNK cells were incubated alone or with phorbol 12-myristate 13-acetate (PMA) and ionomycin (0.5 μg/mL, Sigma-Aldrich), together with CD107a antibody for 4 h. After one hour of incubation, monensin (GolgiStop, BD Biosciences) was added to cultures to inhibit protein transportation. Subsequently, surface staining with CD56 (clone NCAM16.2), CD16 (clone 3G8), CD3 (clone UCHT1) and PD1 (clone EH12.1) was performed for 25 min at 4 °C, in the dark. For intracellular staining of IFN$\gamma$ (clone B27) and TNF (clone MAb11) (all from BD Bioscience) cells were washed with PBS followed by fixation and permeabilization with cytofix/cytoperm (BD Biosciences). Cell were incubated for 30 min at RT with intracellular antibodies. Cells were washed and resuspended in PBS. Acquisition was performed with Beckman Coulter Cytoflex or BD Symphony flow cytometers. Analysis was performed with FlowJo analysis software version 10. Gates were placed on the unstimulated samples for the readout of CD107a, IFNγ and TNF.

### Chromium release assay

NK cell cytotoxicity was measured in a standard ^51^Cr-release assay against tumor target cells. Briefly, target cells were labeled with 100 uL sodium chromate (PerkinElmer) for one hour at 37 °C, after which they were washed three times with PBS. NK cells were mixed with the labeled target cells at different effector to target ratios and incubated for four hours. 20 μL of the supernatant was transferred to LumaPlate-96 and subsequently analyzed with a MicroBeta2 counter (PerkinElmer).

### Live cell imaging assays

Live cell imaging was performed as recently described [25]. Briefly, 3 × 10^4^ PD-L1^+^ 786-O WT or PD-L1^*−*^ 786-O KO cells that were previously transduced to express the fluorescent protein tdTomato were seeded per well in a low-attachment 96-well plate and incubated at 37 °C, 5% CO_2_ for 72 h to allow spheroids to form spontaneously. Prior to analysis, 3 × 10^3^ NK-92 or pNK cells were added to the culture. The number of killed target cells was monitored by imaging every four hours over 48 h (NK-92) to seven days (pNK) using an IncuCyte S3 Live Cell Analysis System (Sartorius). Percent of killing was quantified as decrease in red intensity and normalized to the red fluorescence intensity at the beginning of the assay with the formula $\frac{\textit{red cell count at timepoint x}}{{\textit{red cell count at timepoint 1}}}$.

### Proliferation assays

NK-92 and pNK cells were labeled with Cell Trace Violet (ThermoFisher) according to the manufacturer’s recommendations. Cells were cultured alone or co-cultured in the presence of PD-L1^+^ 786-O WT or PD-L1^*−*^ 786-O KO cells at an effector to target ratio of 1:1. Acquisition was performed with Beckman coulter Cytoflex flow cytometers. Analysis was performed with FlowJo analysis software version 10.

### Statistical analysis

The Student’s t test was used to compare the means of two groups. Two-way ANOVA test was used to compare the means between several groups. *p* < 0.05 was determined as statistically significant (*), *p* < 0.01 (**), *p* < 0.001 (***), *p* < 0.0001 (****) as statistically highly significant. Statistical analysis was performed with GraphPad Prism software version 9 (GraphPad, La Jolla, USA).

## Results

### Expression of PD1-based chimeric switch receptors in NK-92 cells

**Fig. 1:**
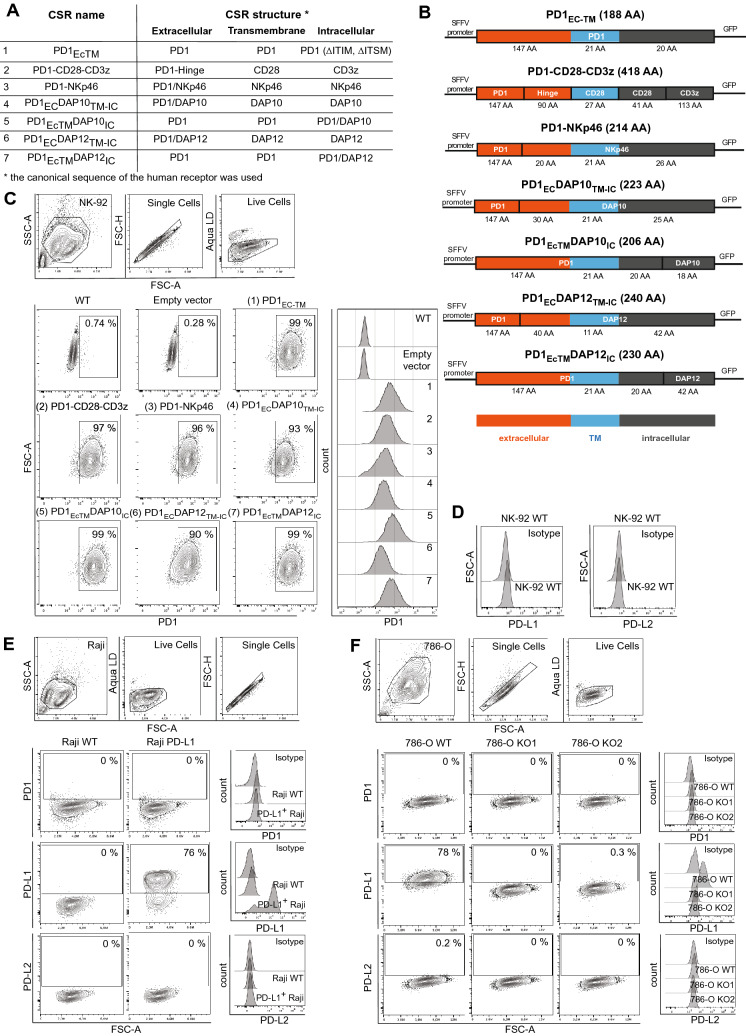
PD1-based chimeric switch receptors are stably expressed in NK-92 cells. **A**, **B** Table and vector maps depicting the design of the truncated PD1 receptor (PD1_EcTM_) and six chimeric switch receptors (CSR) with different signaling domains **C** NK-92 cells containing different PD1-CSR and sorted for positive PD1 surface staining. **D** PD-L1 and PD-L2 expression on NK-92 cells. **E** PD1, PD-L1 and PD-L2 expression on PD-L1^+^ Raji and PD-L1^*−*^ Raji WT cells. **F** PD1, PD-L1 and PD-L2 expression on PD-L1^+^ 786-O WT and PD-L1^*−*^ 786-O KO cells

Initially, six different CSR constructs were generated with the purpose of determining optimal signaling in NK cells. All CSR expressed the unmodified human PD1 extracellular domain fused with various activating intracellular domains. Specifically, DAP10, DAP12, CD3ζ and NKp46 were utilized. Furthermore, a control coding for a truncated, signaling-deficient PD1 construct was generated (Fig. 1A, B). PD1 surface expression among the untransduced or empty vector transduced NK-92 cell lines remained below 2% of the total population. After sorting, all other transduced cell lines stably expressed the transgenes as confirmed by positive PD1 staining. As expected, expression levels differed between the constructs (Fig. 1C). Importantly, neither PD-L1 nor PD-L2 expression was detected in NK-92 wildtype (WT) cells (Fig. 1D).

### Generation of target cell lines

To study the function of the PD1-CSR^+^ NK-92 cell lines, PD-L1^+^ and PD-L1^*−*^ target cell lines were generated. As target cell lines 786-O and Raji cells were chosen, with the first expressing PD-L1 and the latter being devoid of PD-L1. The target cell lines were chosen due to their different potential to activate NK cells. Based on these cell lines, PD-L1 knock-out 786-O (786-O KO) cell lines and a PD-L1^+^ Raji cell line were generated. Hereafter, the target cell lines are referred to as PD-L1^+^ 786-O WT, PD-L1^*−*^ 786-O KO1, PD-L^*−*^ 786-O KO2, PD-L1^+^ Raji and PD-L1^*−*^ Raji WT. Neither of the target cell lines expressed PD1 or PD-L2 (Fig. 1E, F). The genetic modification of the target cell lines did not alter the expression of other ligands for activating NK cell receptors (Figure S1A, B).

### PD1-CSR transduced NK-92 cell lines show superior degranulation and cytokine secretion

**Fig. 2:**
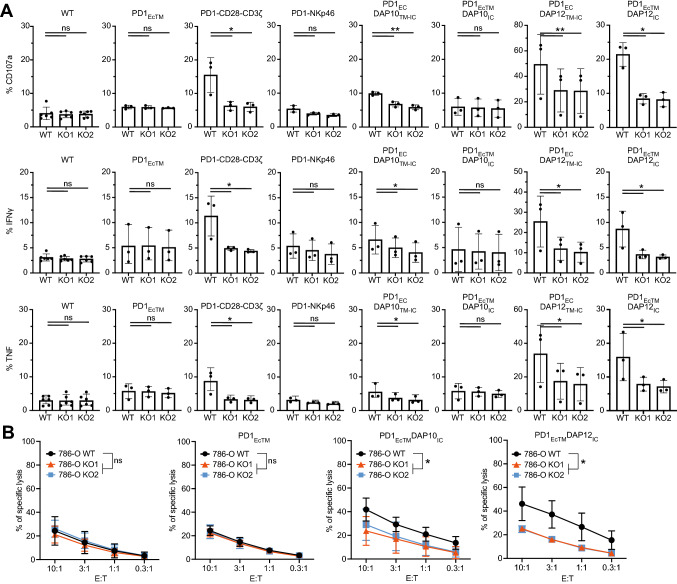
PD1-CSR^+^ NK-92 cells increase degranulation, cytokine production and killing of PD-L1^+^ target cells. **A** Percentage of CD107a, IFNγ and TNF by different PD1-CSR^+^ NK-92 cells against PD-L1^+^ 786-O WT and two PD-L1^*−*^ 786-O KO cell lines. Each data point represents the mean (± SD) of three independent experiments performed in triplicates. **B** Killing of PD-L1^+^ 786-O WT versus PD-L1^*−*^ 786-O KO1 and PD-L1^*−*^ 786-O KO2 cells by NK-92 WT, PD1_EcTM_^+^, PD1_EcTM_DAP10_IC_^+^ or PD1_EcTM_DAP12_IC_^+^ NK-92 cells. Each data point represents the mean (± SD) of three independent experiments performed in quadruplets. Statistical significance (* *p* < 0.05; ** *p* < 0.01) was determined with a two-way ANOVA test

To evaluate the induction of degranulation and cytokine expression, CD107a, IFNγ and TNF expression by all generated PD1-CSR^+^ NK-92 cell lines and controls was assessed against PD-L1^+^ 786-O WT and PD-L1^*−*^ 786-O KO cell lines (Fig. 2A, Figure S2A). Generally, 786-O cells are resistant to NK cell-mediated cytotoxicity and the PD1-CSR^+^ NK-92 cells were tested for their ability to circumvent the resistance. In line with this, CD107a, IFNγ and TNF expression by NK-92 WT and PD1_EcTM_^+^ NK-92 remained below 10% against both PD-L1^+^ 786-O WT and PD-L1^*−*^ 786-O KO cell lines, with no significant differences between the three target cell lines.

The PD1-CD28-CD3ζ and PD1_EcTM_DAP12_IC_ transduced NK-92 cell lines showed a three-to four-fold increase in CD107a expression and corresponding increase in IFNγ and TNF expression against PD-L1^+^ 786-O WT compared to both PD-L1^*−*^ 786-O KO cell lines. CD107a, IFNγ and TNF production was 1.5-fold higher by PD1_EC_DAP10_TM-IC_^+^ NK-92 against PD-L1^+^ 786-O WT cells. CD107a, IFNγ and TNF expression by PD1_EC_DAP12_TM-IC_^+^ NK-92 cells showed the highest degranulation against PD-L1^+^ 786-O WT cells among the PD1-CSR^+^ NK-92 cell lines, approaching 50% (± 12%), 30% (± 8.5%) and 30% (± 8.5%), respectively. Notably, PD1-NKp46 and PD1_EcTM_DAP10_IC_ expressing NK-92 cell lines did not show increased CD107a nor IFNγ or TNF expression against PD-L1^+^ 786-O WT compared to PD-L1^*−*^ 786-O KO cell lines. To conclude, PD1-CD28-CD3ζ^+^, PD1_Ec_DAP10_TM-IC_^+^, PD1_Ec_DAP12_TM-IC_^+^ and PD1_EcTM_DAP12_IC_^+^ NK-92 cells increased degranulation and cytokine expression against PD-L1^+^ 786-O WT cells which are inherently resistant to NK cell-mediated cytotoxicity.

### PD1-CSR transduced NK-92 cell lines show superior degranulation and cytokine secretion against PD-L1^+^ Raji cells

To extend these results to a cell line that is already highly susceptible to NK cell killing, all generated PD1-CSR^+^ NK-92 cell lines were assessed against PD-L1^+^ Raji cells and PD-L1^*−*^ Raji WT cells (Figure S2 B–D). NK92 WT and PD1_EcTM_^+^ NK92 cells showed a high CD107a, IFNγ and TNF expression against both PD-L1^*−*^ Raji WT cells and PD-L1^+^ Raji cells with values reaching up to 80% (± 7%), 70% (± 6%) and 70% (± 1.2%), respectively. Despite the high baseline values, the expression of CD107a, IFNγ and TNF by PD1-CD28-CD3ζ^+^, PD1_EcTM_DAP10_IC_^+^, PD1_Ec_DAP12_TM-IC_^+^ and PD1_EcTM_DAP12_IC_^+^ NK-92 cells was significantly higher against PD-L1^+^ Raji cells compared to PD-L1^*−*^ Raji WT cells. PD1-NKp46 and PD1_Ec_DAP10_TM-IC_ expressing NK-92 cell lines did not increase CD107a, IFNγ or TNF expression against PD-L1^+^ Raji cells compared to PD-L1^*−*^ Raji WT cells. Taken together, PD1-CD28-CD3ζ^+^, PD1_EcTM_DAP10_IC_^+^, PD1_Ec_DAP12_TM-IC_^+^ and PD1_EcTM_DAP12_IC_^+^ NK-92 cells increased degranulation and cytokine expression against PD-L1^+^ Raji cells.

### Receptor-independent degranulation and cytokine production by PD1-CSR^+^ NK-92 cells

To confirm that the induction of CD107a, IFNγ and TNF expression is based on PD1-PD-L1 interaction, degranulation and cytokine expression by unstimulated or maximal chemically (PMA/Iono) stimulated PD1-CSR^+^ NK-92 cell lines was measured (Figure S3A–C). No significant differences in CD107a, IFNγ or TNF expression by unstimulated PD1-CSR^+^ NK-92 cell lines was observed. However, chemical stimulation of PD1-CD28-CD3ζ^+^ and PD1-NKp46^+^ NK-92 cells resulted in reduced CD107a, but not IFNγ or TNF expression, compared to NK-92 WT cells. The other PD1-CSR^+^ NK-92 cells did not show differences in CD107a, IFNγ or TNF expression after chemical stimulation compared to NK-92 WT cells. PD1 positivity decreased in the PD1-NKp46^+^ NK-92 cell line in the absence of stimulation as well as after chemical stimulation or target cell recognition. After chemical stimulation or recognition of PD-L1^+^ target cells, PD1 surface expression also decreased in the PD1_Ec_DAP12_TM-IC_ expressing cell line and remained stable for the other PD1-CSR^+^ NK-92 cell lines (Figure S3D, E). Taken together, PD1_EcTM_DAP10_IC_^+^ and PD1_EcTM_DAP12_IC_^+^ NK-92 cells showed a stable PD1 surface staining, a higher CD107a, IFNγ and TNF expression upon PD-L1^+^ target cell recognition and no alteration in degranulation or cytokine secretion in the unstimulated or maximal chemically stimulated controls compared to NK-92 WT cells. Genetic modification did not alter the expression of other activating and inhibitory NK cell receptors on mock-transduced, PD1_EcTM_, PD1_EcTM_DAP10_IC_ and PD1_EcTM_DAP12_IC_ transduced NK92 cells (Figure S4A) Therefore, PD1_EcTM_DAP10_IC_ and PD1_EcTM_DAP12_IC_ CSR were chosen for the following assays.

### PD1_EcTM_DAP10_IC_^+^ and PD1_EcTM_DAP12_IC_^+^NK-92 cells show higher killing of PD-L1^+^ 786-O WT cells

Since PD1_EcTM_DAP10_IC_^+^ and PD1_EcTM_DAP12_IC_^+^ NK-92 cells showed higher efficacy, we explored whether these cells would also directly kill tumor target cells. Killing of ^51^Cr labeled PD-L1^+^ 786-O WT or PD-L1^*−*^ 786-O KO cells by either NK-92 WT, PD1_EcTM_^+^, PD1_EcTM_DAP10_IC_^+^ or PD1_EcTM_DAP12_IC_^+^ NK-92 cell lines was assessed at different effector to target (E:T) ratios (Fig. 2B). There was no significant difference in killing of PD-L1^+^ 786-O WT cells compared to both PD-L1^*−*^ 786-O KO cell lines by either NK-92 WT or PD1_EcTM_^+^ NK92 cells. PD1_EcTM_DAP10_IC_^+^ NK-92 cells increased killing of PD-L1^+^ 786-O WT cells twofold compared to both PD-L1^*−*^ 786-O KO cell lines. Similarly, PD1_EcTM_DAP12_IC_^+^ NK-92 cells increased killing of PD-L1^+^ 786-O WT cells by twofold at higher E:T ratios and 3.5-fold at lower E:T ratios compared to PD-L1^*−*^ 786-O KO cells. No significant difference in killing of PD-L1^+^ Raji compared to PD-L1^*−*^ Raji WT cells was observed by either of the PD1-CSR^+^ NK-92 cells (Figure S4 B-E).

### PD1_EcTM_DAP10_IC_^+^ and PD1_EcTM_DAP12_IC_^+^NK-92 show increased killing of large PD-L1^+^ 786-O WT tumor spheroids

**Fig. 3:**
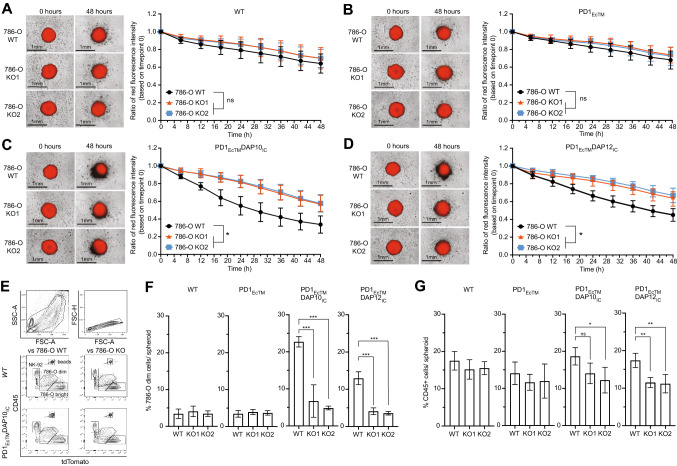
PD1-CSR^+^ NK-92 cells increase cytotoxicity against PD-L1^+^ tumor spheroids. **A**–**D** Killing of PD-L1^+^ 786-O WT versus PD-L1^*−*^ 786-O KO1 and PD-L1^*−*^ 786-O KO2 tumor spheroids by NK-92 WT, PD1_EcTM_^+^, PD1_EcTM_DAP10_IC_^+^ or PD1_EcTM_DAP12_IC_^+^ NK-92 cells. Each data point represents the mean (± SD) of six independent experiments performed in duplicates. **E–G** Tumor spheroids were collected, washed, dissociated and analyzed by flow cytometry. Displayed is the gating strategy (**E**), the percentage of 786-O^dim^ cells per spheroid (**F**) and percentage of CD45^+^ NK-92 cells per spheroid (**G**). Each data point represents the mean (± SD) of three independent experiments performed in duplicates. Statistical significance (* *p* < 0.05; ** *p* < 0.01; *** *p* < 0.001) was determined with a two-way ANOVA test

Since 786-O cells can form large tumor spheroids with diameters reaching up to one millimeter, the ability of PD1_EcTM_DAP10_IC_^+^ and PD1_EcTM_DAP12_IC_^+^ NK-92 cells to kill PD-L1^+^ 786-O WT cells was tested in the 3D co-culture model as it more accurately resembles the TME than a 2D co-culture model. For this purpose, PD-L1^+^ 786-O WT and both PD-L1^*−*^ 786-O KO cell lines were further transduced to express the red fluorescence protein tdTomato. Killing of 786-O spheroids was assessed based on the decrease of red fluorescence intensity as captured with the IncuCyte live cell imager (Fig. 3A–D; supplemental Video V1-V4) and by flow cytometry (Fig. 3E–F). Red fluorescence intensity decreased from 100 to 70% (± 11%) in PD-L1^+^ 786-O WT and PD-L1^*−*^ 786-O KO cell lines over a 48 h period when NK-92 WT or PD1_EcTM_^+^ NK-92 cells were added to the tumor spheroids, with no significant differences between the three target cell lines. PD1_EcTM_DAP10_IC_^+^ NK-92 cells resulted in a reduction of red fluorescence intensity from 100 to 35% (± 10%) in the PD-L1^+^ 786-O WT cells and from 100 to 60% (± 9%) in the PD-L1^*−*^ 786-O KO cell lines, with statistical significance between the PD-L1^+^ and PD-L1^*−*^ target cell lines. Similarly, there was a significant decrease in red fluorescence intensity in PD-L1^+^ 786-O WT cells (100% to 45% (± 7%)) compared to PD-L1^*−*^ 786-O KO cell lines (100% to 65% (± 8%)) when PD1_EcTM_DAP12_IC_^+^ NK-92 cells were added. The tumor spheroids were therafter harvested, washed and dissociated to assess spheroid killing by flow cytometry (Fig. 3E–F; Figure S5 A). Live 786-O cells showed a high expression of tdTomato and were classified as 786-O^bright^ while dying 786-O cells gradually lost tdTomato expression and were classified as 786-O^dim^ cells. As expected, both PD1_EcTM_DAP10_IC_^+^ and PD1_EcTM_DAP12_IC_^+^ NK-92 cells, but not NK-92 WT or PD1_EcTM_^+^ NK-92 cells, led to a significant increase in the 786-O^dim^ population and corresponding decrease in the 786-O^bright^ population against the PD-L1^+^ 786-O WT but not PD-L1^*−*^ 786-O KO cell lines. Furthermore, the percentage and total amount of CD45^+^ cells within the 786-O tumor spheroids was assessed (Fig. 3G, Figure S5 B). There was a statistically significant difference in the percentage of CD45^+^ cells within the PD-L1^+^ 786-O WT spheroid compared to the PD-L1^*−*^ 786-O KO spheroids when PD1_EcTM_DAP10_IC_^+^ and PD1_EcTM_DAP12_IC_^+^ NK-92 cells were added. However, no statistical significant difference was observed for the absolute numbers of CD45^+^ cells. Lastly, proliferation of PD1-CSR^+^ NK-92 cells was measured. No significant differences in proliferation of PD1_EC-TM_DAP10_IC_^+^ or PD1_EC-TM_DAP12_IC_^+^ NK-92 cells compared to WT or PD1_EC-TM_^+^ NK-92 cells were observed when cultured alone (Figure S5C), exposed one-time to PD-L1^+^ 786-O WT cells (Figure S5D) or repetitively exposed to PD-L1^+^ 786-O WT cells (Figure S5E). In summary, both PD1_EC-TM_DAP10_IC_^+^ and PD1_EC-TM_DAP12_IC_^+^ NK-92 cells increased killing of large PD-L1^+^ 786-O WT tumor spheroids over a 48 h period.

### PD1_EcTM_DAP10_IC_^+^ and PD1_EcTM_DAP12_IC_^+^ pNK cells increase degranulation and cytokine expression against PD-L1^+^ Raji cells

**Fig. 4:**
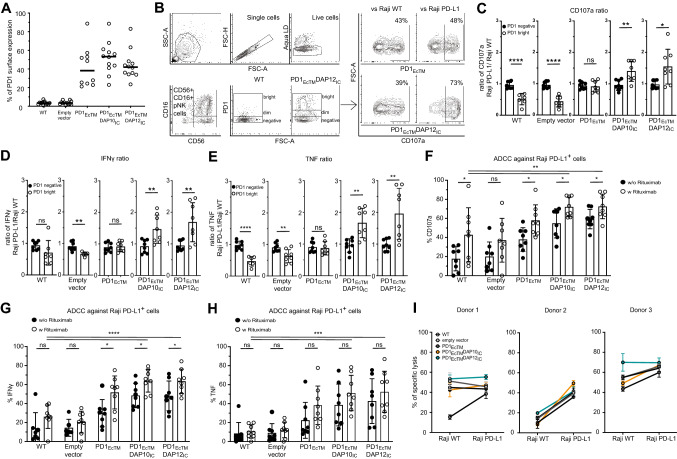
PD1-CSR^+^ pNK cells increase degranulation and cytokine production against PD-L1^+^ target cells. **A** PD1 surface expression on untransduced or transduced CD56^+^ CD3^*−*^ pNK cells. Each dot represents PD1 surface expression on one individual donor (*n* = 12). The mean of all 12 donors is displayed. **B** Gating strategy for degranulation assays. WT and empty vector transduced cells were gated on PD1^dim^ population while PD1-CSR^+^ cells were gated on the PD1^high^ population. **C**–**E** Ratio of CD107a (**C**), IFNγ (**D**) and TNF (**E**) expression by different PD-CSR^+^ pNK cells against PD-L1^+^ Raji cells versus PD-L1^*−*^ Raji WT cells. Each dot represents the mean of one individual donor, performed in duplicates (*n* = 8) The mean ± SD of all 8 donors is displayed. **F–H** Percentage of CD107a (**F**), IFNγ (**G**) and TNF (**H**) expression by different PD-CSR^+^ pNK cells against PD-L1^+^ Raji cells. Each dot represents the mean of one individual donor, performed in duplicates (*n* = 8) The mean ± SD of all 8 donors is displayed. **I** Killing of PD-L1^+^ Raji cells versus PD-L1^*−*^ Raji WT cells by different PD-CSR^+^ pNK cells at an E:T of 1:1. Displayed are data from 3 independent donors with each data point representing the mean (± SD) of one experiment performed in triplicates. Statistical significance was determined with a Students t test (* *p* < 0.05, ** *p* < 0.01, ***p < 0.001, ****p < 0.0001)

With the aim to implement PD1-CSR for adoptive cell therapies, the function of PD1_EcTM_DAP10_IC_ and PD1_EcTM_DAP12_IC_ constructs in pNK cells, isolated from healthy donor PBMCs, was tested. While PD1 surface expression in untransduced pNK cells or mock-transduced (empty vector) pNK cells remained below 5%, its expression increased on average to 42% (± 22%), 52% (± 17%) and 45% (± 16%) in the pNK cells transduced with either PD1_EcTM_, PD1_EcTM_DAP10_IC_ or PD1_EcTM_DAP12_IC_ CSR, respectively (Fig. 4A). Two separate levels of PD1 surface expression were observed and CD56^+^ CD16^+^ pNK cells were classified as either PD1^dim^ or PD1^bright^ (Fig. 4B). The expression of the main activating and inhibitory NK cell receptors on PD1^+^ PD1-CSR^+^ pNK cells compared to PD1^+^ WT or mock-transduced pNK cells from three different donors was measured by flow cytometry (Figure S6A). Although inter-individual differences in receptor expression were observed, the genetic modification of pNK cells with PD1-CSR did not cause any consistent intra-individual phenotypic changes of pNK cells. CD107a, IFNγ and TNF expression were measured in a degranulation assay against PD-L1^+^ Raji cells and PD-L1^*−*^ Raji WT cells (Fig. 4C–E, Figure S7, Figure S8A–C). To facilitate direct comparison, the fold ratio of CD107a, IFNγ and TNF expression against PD-L1^+^ Raji cells compared to PD-L1^*−*^ Raji WT cells is displayed, with numbers below one indicating a reduction and numbers above one an increase upon PD-L1 engagement in the respective parameter (Fig. 4C–E). PD1^dim^ pNK cells from WT or mock-transduced pNK cells showed a lower CD107a expression against PD-L1^+^ Raji cells, significantly reducing the ratio below one. On the contrary, PD1^bright^ PD1_EcTM_^+^ pNK cells increased degranulation against PD-L1^+^ Raji cells compared to WT or mock-transduced pNK cells, raising the ratio to one. PD1^bright^ PD1_EcTM_DAP10_IC_^+^ and PD1^bright^ PD1_EcTM_DAP12_IC_^+^ pNK cells significantly increased CD107a expression against PD-L1^+^ Raji cells compared to PD-L1^−^ Raji WT cells, raising the ratio to 1.5. Similarly, the ratio of IFNγ and TNF expression against PD-L1^+^ Raji cells compared to PD-L1^*−*^ Raji WT cells was higher than one for the PD1^bright^ PD1_EcTM_DAP10_IC_^+^ and PD1^bright^ PD1_EcTM_DAP12_IC_^+^ pNK cells but not PD1^bright^ PD1_EcTM_^+^ pNK cells or PD1^dim^ WT or PD1^dim^ mock-transduced pNK cells. In conclusion, PD1_EcTM_^+^ pNK cells blocked native PD1-PD-L1 mediated pNK cell inhibition, while both PD1_EcTM_DAP10_IC_^+^ and PD1_EcTM_DAP12_IC_^+^ pNK cells reverted inhibition into an increased degranulation and cytokine expression against PD-L1^+^ Raji cells.

### PD1_EcTM_DAP10_IC_^+^ and PD1_EcTM_DAP12_IC_^+^ pNK cells increase degranulation and cytokine expression against PD-L1^+^ Raji cells together with ADCC

After demonstrating functionality of PD1_EcTM_DAP10_IC_ and PD1_EcTM_DAP12_IC_ CSR in pNK cells, their ability to synergize with CD16 mediated ADCC was tested to evaluate their potential in combinatorial treatment approaches. The percentages of CD107a, IFNγ and TNF expression against PD-L1^+^ Raji cells and PD-L1^*−*^ Raji WT cells with or without the addition of the anti-CD20 mAb Rituximab were measured (Fig. 4F–H, Figure S7). Rituximab increased CD107a, IFNγ and TNF expression against PD-L1^+^ Raji cells in both PD1^dim^ WT and mock-transduced pNK cells compared to no ADCC. Likewise, PD1^bright^ PD_EcTM_^+^, PD1_EcTM_DAP10_IC_^+^ and PD1_EcTM_DAP12_IC_^+^ pNK cells showed increased CD107a, IFNγ and TNF expression with the addition of Rituximab compared to no ADCC. Overall, PD1^bright^ PD1_EcTM_DAP10_IC_^+^ and PD1_EcTM_DAP12_IC_^+^ pNK showed the highest increase in CD107a, IFNγ and TNF expression with values reaching up to 72% (± 12%), 63% (± 12%) and 51% (± 19%), respectively, compared to WT or mock-transduced pNK cells with values approaching 42% (± 28), 26% (± 12) and 11% (± 8%), respectively. Compared to PD1^bright^ PD1-CSR^+^ pNK cells, PD1^dim^ PD1_EcTM_DAP10_IC_^+^ and PD1_EcTM_DAP12_IC_^+^ pNK cells did not increase degranulation and cytokine production against PD-L1^+^ Raji cells but equalized it to the CD107a, IFNγ and TNF expression levels against PD-L1^*−*^ Raji cells (Figure S8A–C). In conclusion, both PD1_EcTM_DAP10_IC_^+^ and PD1_EcTM_DAP12_IC_^+^ pNK cells increased degranulation and cytokine expression against PD-L1^+^ Raji cells with or without the addition of Rituximab and thus reverted native PD1 mediated NK cell inhibition.

### PD1_EcTM_DAP12_IC_^+^ pNK cells increase degranulation and cytokine expression against PD-L1^+^ 786-O WT cells

To extend these results, degranulation and cytokine expression of PD1-CSR^+^ pNK cells was also measured against PD-L1^+^ 786-O WT and PD-L1^*−*^ 786-O KO cells (Figure S8D–F). Similar to the data from NK92 cell lines, WT or mock-transduced pNK cells showed a low expression of CD107a, IFNγ and TNF against both PD-L1^+^ 786-O WT and PD-L1^*−*^ 786-O KO cells, with no significant differences between the target cell lines. Compared to that PD1^bright^ PD1_EcTM_DAP12_IC_^+^ pNK cells, but not PD1^dim^ PD1_EcTM_DAP12_IC_^+^ pNK cells, significantly increased CD107a, IFNγ and TNF expression against PD-L1^+^ 786-O WT cells compared to PD-L1^*−*^ 786-O KO cells. While PD1_EcTM_^+^ and PD1_EcTM_DAP10_IC_^+^ pNK cells did not correlate with a higher CD107a expression, they showed a higher IFNγ and TNF expression against both PD-L1^+^ 786-O WT and PD-L1^*−*^ 786-O KO cells. All in all, PD1^bright^ PD1_EcTM_DAP12_IC_^+^ pNK cells increased degranulation and cytokine expression against PD-L1^+^ 786-O WT cells.

### PD1_EcTM_DAP10_IC_^+^ and PD1_EcTM_DAP12_IC_^+^ pNK cells do not alter killing of PD-L1^+^ target cells

Next, the ability of the PD1-CSR enriched pNK cells to kill PD-L1^+^ and PD-L1^*−*^ target cells in a 2D and 3D co-culture model was assessed. No difference in killing of PD-L1^+^ Raji cells compared to PD-L1^*−*^ Raji WT cells by PD1_EcTM_DAP10_IC_^+^ or PD1_EcTM_DAP12_IC_^+^ pNK cells from three different donors compared to WT, mock-transduced or PD1_EcTM_^+^ pNK cells was observed (Fig. 4I, Figure S9A). Similarly, neither PD1_EcTM_DAP10_IC_^+^ nor PD1_EcTM_DAP12_IC_^+^ pNK cells increased killing of PD-L1^+^ 786-O WT tumor spheroids compared to PD-L1^*−*^ 786-O KO tumor spheroids (Figure S9B). However, this lack of killing ability might be due to the fact that pNK cells were not sorted for high expression of PD1-CSR prior to use. Finally, the proliferative capacity of PD1-CSR^+^ pNK cells was measured (Figure S9C). PD1^dim^ WT pNK cells showed a lower proliferation rate compared to PD1^negative^ WT pNK cells. In contrast, PD1^bright^ PD1-CSR^+^ pNK cells increased the proliferation rate above the value observed in PD1^dim^ WT pNK cells closer to the value observed in PD1^negative^ pNK cells. All in all, pNK cells, enriched with PD1_EcTM_DAP10_IC_^+^ and PD1_EcTM_DAP12_IC_^+^ pNK cells, did not show an increased killing of PD-L1^+^ tumor target cells.

### PD1_EcTM_DAP10_IC_^+^ and PD1_EcTM_DAP12_IC_^+^ pNK cells from patients with newly diagnosed MM increase degranulation and cytokine production against autologous PD-L1^+^ tumor samples

**Fig. 5:**
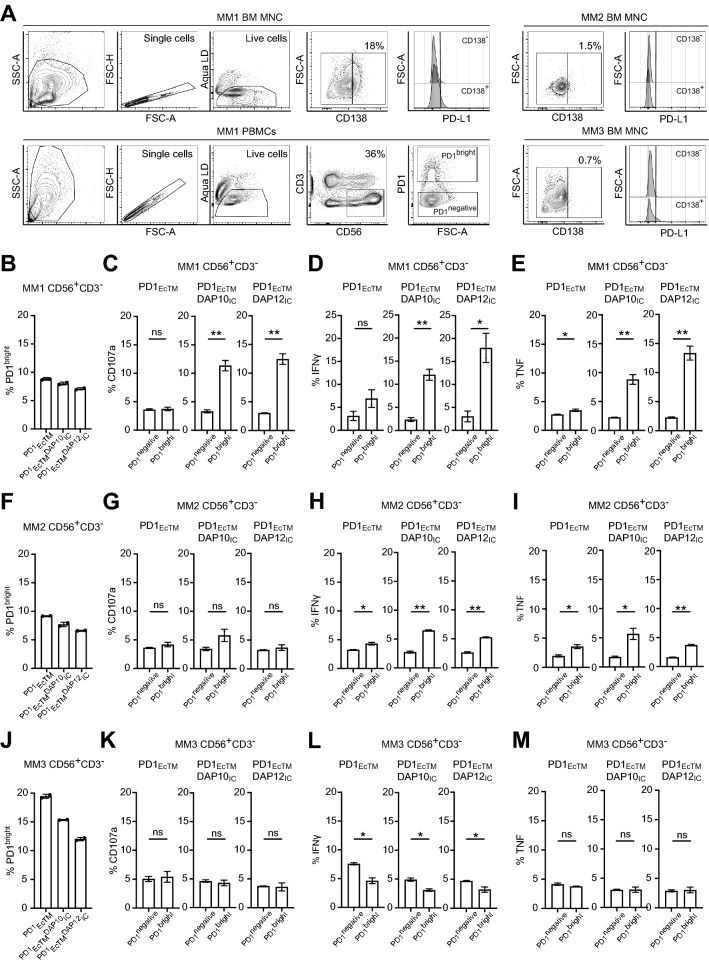
PD1-CSR^+^ pNK cells increase degranulation and cytokine production against PD-L1^+^ autologous tumor samples. **A** Flow cytometry plots show the gating strategy of BM MNC (upper panel) and PBMCs (lower panel) from newly diagnosed MM patients **B** Percentage of PD1^bright^ cells among CD56^+^CD3^−^ pNK cells from MM donor 1 **C**–**E** Percentage of CD107a (**C**), IFNγ (**D**) and TNF (**E**) by different PD1^negative^ versus PD1^bright^ PD1-CSR^+^ pNK cells from donor 1 against autologous BM MNC. **F** Percentage of PD1^bright^ cells among CD56^+^CD3^−^ pNK cells from MM donor 2 **G**–**I** Percentage of CD107a (**G**), IFNγ (**H**) and TNF (**I**) by different PD1^negative^ versus PD1^bright^ PD-CSR^+^ pNK cells from donor 2 against autologous BM MNC. **J** Percentage of PD1^bright^ cells among CD56^+^CD3^−^ pNK cells from MM donor 3K–**M**) Percentage of CD107a (**K**), IFNγ (**L**) and TNF (**M**) by different PD1^negative^ versus PD1^bright^ PD-CSR^+^ pNK cells from donor 3 against autologous BM MNC. Displayed are data from each donor with each data point representing the mean (± SD) of one experiment performed in duplicates. Statistical significance was determined with a Students t test (* *p* < 0.05, ** *p* < 0.01)

After establishing that both PD1_EcTM_DAP10_IC_^+^ and PD1_EcTM_DAP12_IC_^+^ pNK cells from healthy donors increased degranulation and cytokine expression against PD-L1^+^ tumor cell lines, their function in pNK cells from patients with MM against autologous bone marrow mononuclear cells (BM MNC) was evaluated. For this, PBMCs from three patients with newly diagnosed MM were expanded for 13 days prior to transduction with lentiviral vectors, encoding PD1_EcTM_, PD1_EcTM_DAP10_IC_ and PD1_EcTM_DAP12_IC_ CSR. Degranulation was performed on day 4 after transduction with approximately 40% CD56^+^CD3^*−*^ pNK cells among the expanded PBMCs (Fig. 5A). CD138 expression on BM MNC, indicative of malignant plasma cells, was measured by flow cytometry and only detected in BM MNC from donor 1 (MM1 BM MNC), but not donor 2 (MM2 BM MNC) or donor 3 (MM3 BM MNC). A more detailed phenotypic analysis of BM MNC from donor 1 and donor 2 is provided in Figure S10. PD-L1 and PD-L2 expression was detected on CD138^+^ cells, but only at very low levels on CD138^*−*^ BM MNCs (Fig. 5A, Figure S10A, B). The percentage of PD1^bright^ cells on CD56^+^CD3^*−*^ pNK cells ranged between 7 to 20% depending on the CSR construct and donor (Fig. 5B, F, J). Both PD1^bright^ PD1_EcTM_DAP10_IC_^+^ and PD1_EcTM_DAP12_IC_^+^ pNK cells from donor 1 increased CD107a, IFNγ and TNF expression significantly against autologous BM MNC two-to three-fold compared to PD1^negative^ pNK cells (Fig. 5C–E). PD1^bright^ PD1_EcTM_DAP10_IC_^+^ and PD1_EcTM_DAP12_IC_^+^ pNK cells from donor 2 did not increase CD107a expression, but showed an increased IFNγ and TNF expression compared to PD1^negative^ pNK cells (Fig. 5G–I). PD1^bright^ PD1_EcTM_DAP10_IC_^+^ and PD1_EcTM_DAP12_IC_^+^ pNK cells from donor 3 did not increase CD107a, IFNγ or TNF expression, but showed a slight decreased IFNγ response (Fig. 5K–M). These data confirm that PD1_EcTM_DAP10_IC_^+^ and PD1_EcTM_DAP12_IC_^+^ pNK augment degranulation and cytokine expression against autologous CD138^+^ PD-L1^+^ malignant bone marrow cells.

## Discussion

In this paper, we demonstrate that PD1-based CSR revert NK cell inhibition imposed by PD1-PD-L1 engagement and, hence, are able to skew the response toward NK cell activation. The results emphasize that replacement of the ITIM and ITSM domain of PD1 by either an ITAM or YINM motif confers a higher degranulation and cytokine production by both NK-92 and pNK cells toward PD-L1 expressing target cells in 2D and 3D tumor co-culture models. Most importantly, pNK cells from patients with MM were successfully transduced to express PD1_EcTM_DAP10_IC_ or PD1_EcTM_DAP12_IC_ CSR and showed higher degranulation and cytokine expression against autologous CD138^+^PD-L1^+^ tumor samples.

Different to CAR, the present CSR shall enhance NK cell cytotoxicity in concert with general target cell recognition and tip the balance toward activation in an immunosuppressive TME. The aim in the present study was to design CSR that are not activating NK cells toward healthy tissue where PD1 ligands are abundantly expressed and can explain common side-effects of immune-checkpoint blockade with mAb [26]. Therefore, the human canonical sequence of PD1 without any further modification was employed. Furthermore, only one signaling domain was used compared to the second and third generation CARs that are designed with different co-stimulatory domains. Whether PD-L1 targeting CAR NK cells would, however, cause severe side-effects is yet not elucidated. PD-L1 targeting high-affinity NK-92 cells (PD-L1-t-haNK) showed promising preclinical results and are currently in early phase clinical trials (NCT04050709, NCT04847466, NCT04927884) [27]. The results and toxicity profile of these PD-L1 CAR expressing NK-92 cells are eagerly awaited. However, employing the extracellular domain of PD1, instead of the single-chain fragment targeting PD-L1, poses the advantage of recognizing both PD-L1 and PD-L2. In humans, PD-L2 is mainly expressed on professional antigen-presenting cells and over-expressed in cancer cells as well as stromal and epithelial cells of several tumor types [28]. Targeting both PD1 ligands was associated with a better clinical outcome in lung cancer [29]. Besides PD1, NK cells express a plethora of canonical checkpoints that are important both for control of activation as well as retention of educated state upon adoptive transfer [30, 31]. The surface retained checkpoints include NKG2A, T cell immunoreceptor with Ig and ITIM domains (TIGIT), Lymphocyte Activating Gene 3 (LAG3), T cell immunoglobulin domain and mucin domain 3 (TIM3) as well as inhibitory KIRs. It is conceivable that some of these receptors could also be engineered in a similar fashion as described here for PD1.

PD1-CSR^+^ pNK cells from MM patient number 3 showed a decrease in IFNγ production against autologous BM MNCs. Unfortunately, we were not able to determine the factors leading to this small but significant reduction in cytokine expression. Taken the complexity of the immunosuppressive TME into account, it is conceivable that PD1-CSR expressing NK cells might be inhibited by other soluble or receptor-mediated factors. A solution could be the combination of PD1-CSR^+^ pNK cells with other immune checkpoint targeting therapies. Monalizumab, a monoclonal antibody against NKG2A that is widely used in clinical trials, promoted both NK and CD8^+^ T cell anti-cancer functions, especially in combination with PD1-PD-L1 blockade [30]. In line with this, disruption of NKG2A in primary NK cells improved NK cell cytotoxicity against primary MM cells [32].

Blockade of immune checkpoint receptors such as PD1 or TIGIT with mAb was shown to restore NK cell effector functions against tumor cells [15, 33]. However, the majority of available antibodies merely blocks the PD1-PD-L1 interaction and does not induce ADCC to enhance NK cell functions. So far, avelumab is the only PD-L1-targeting antibody available with ADCC function [34]. To date, no clinical study evaluated the combination of avelumab with adoptive NK cell therapy. Here, we show that both PD1_EcTM_DAP10_IC_ and PD1_EcTM_DAP12_IC_ revert PD1 based NK cell inhibition, with PD1_EcTM_DAP12_IC_^+^ cells eliciting a higher increase. In line with our present findings, a PD1-NKG2D CSR with 4-1BB costimulatory domain enhanced killing of PD-L1^+^ target cells, but did not increase cytokine release [35]. NKG2D is a type-II transmembrane protein that dimerizes and forms a hexameric structure with four DAP10 molecules [36]. Moreover, a DAP12 based CAR increased both target cell killing and IFNγ production by pNK cells [37]. The effector cell functionality of CAR-DAP12 transduced NK cells was higher than CAR-CD3ζ transduced cells. Both PD1-CSR constructs were able to revert NK cell hypofunctionality induced by native PD1-PD-L1 signaling. However, this increase was only observed in transduced PD1^bright^ pNK cells. In contrast, PD1^dim^ cells blocked native PD1-PD-L1 engagement and restored degranulation and cytokine secretion. With 5–10% of PD1^bright^ cells within the NK cell product, we have not observed an overall higher target cell killing.

The present findings indicate that PD1-CSR^+^ pNK cells could be employed in combinatorial treatment approaches such as in combination with mAb. Therefore, the ability of PD1-CSR^+^ pNK to engage in ADCC was studied and demonstrated that both PD1_EcTM_DAP10_IC_^+^ and PD1_EcTM_DAP12_IC_^+^ synergistically increased degranulation against PD-L1^+^ Raji cells in combination with Rituximab. Another mAb that is known to work mainly via ADCC is Daratumumab that targets CD38 expressed on malignant plasma cells [38]. Daratumumab is approved as a frontline therapy in patients with newly diagnosed MM [39]. Furthermore, NK cell based therapies are currently in early-phase clinical trials for MM (NCT04558853, EudraCT: 2020-000994-26) [40, 41]. The results of these trials are eagerly awaited. We envision an indication for PD1-CSR^+^ pNK cells in patients with MM, a disease in which immune checkpoint blockade with mAb has failed. Monotherapy with the monoclonal PD1 antibody nivolumab in heavily pretreated MM patients only led to a stable disease without significant disease regression [42]. Two phase III clinical trials, studying the combinatorial application of PD1 receptor blockade by pembrolizumab with an immunomodulatory drug (IMiD) and dexamethasone (Keynote-183, Keynote-185), had to be suspended in 2017 due to dissatisfactory interim results, revealing increased death rates among patients that were enrolled in the experimental arm [43, 44]. Specifically, severe cardiac events, myocarditis and pneumonia were higher in the group that received pembrolizumab, causing increased death rates. Studies are ongoing to determine patient cohorts, combination regimens and treatment agents to efficiently target the PD1-PD-L1 axis in MM and improve patient outcome. Recently, avelumab showed a good toxicity profile but unfortunately no clinical benefit in combination with radiotherapy for relapsed or refractory MM [45]. Promisingly, our preclinical data show that both PD1_EcTM_DAP10_IC_^+^ and PD1_EcTM_DAP12_IC_^+^ pNK cells from newly diagnosed MM patients increase degranulation and cytokine production against autologous PD-L1^+^ CD138^+^ BM MNC while sparing PD-L1^*−*^ CD138^*−*^ samples. However, PD1-CSR^+^ pNK cells could potentially also target PD-L1 expressed on other cells of the TME such as myeloid-derived suppressor cells (MDSC) or tumor-associated macrophages (TAM) and thus re-shape the TME via increased cytokine expression or reduction of pro-tumorigenic cell numbers. Further studies to advance PD1-CSR^+^ pNK cells for the treatment of MM; e.g., in combination with Daratumumab, are warranted. Specifically, PD1-CSR should be tested in pNK cells from a larger cohort of patients with MM to confirm our observations reported here.

In conclusion, we have here demonstrated that PD1_EcTM_DAP10_IC_^+^ and PD1_EcTM_DAP12_IC_^+^ CSR revert PD1-PD-L1 induced NK cell inhibition. PD1-CSR^+^ NK cells hence represent a feasible approach for future adoptive NK cell-based immunotherapy platforms in human cancer treatment.

## Supplementary Information

Below is the link to the electronic supplementary material.

Supplementary file1 (EPS 1871 KB)

Supplementary file2 (EPS 3592 KB)

Supplementary file3 (EPS 2850 KB)

Supplementary file4 (EPS 2469 KB)

Supplementary file5 (EPS 2505 KB)

Supplementary file6 (EPS 3130 KB)

Supplementary file7 (EPS 4184 KB)

Supplementary file8 (EPS 3717 KB)

Supplementary file9 (EPS 3281 KB)

Supplementary file10 (EPS 2499 KB)

Supplementary file11 (MP4 2227 KB)

Supplementary file12 (MP4 2176 KB)

Supplementary file13 (MP4 2257 KB)

Supplementary file14 (MP4 2242 KB)

## Data Availability

All data relevant to the study are included in the article or uploaded as supplementary information.

## Abbreviations

ADCC: Antibody-dependent cellular cytotoxicity
BM MNC: Bone marrow mononuclear cells
CAR: Chimeric antigen receptor
CSR: Chimeric switch receptor
ICI: Immune checkpoint inhibition
ITAM: Immunoreceptor tyrosine-based motif
mAb: Monoclonal antibody
MM: Multiple myeloma
NK: Natural killer
pNK: Primary natural killer
PD1: Programmed death protein 1
PD-L1: Programmed death ligand 1
PD-L2: Programmed death ligand 2
TCR: T cell receptor
TME: Tumor microenvironment
